# Safety-Constrained Reinforcement Learning for Energy-Aware Transmission Scheduling in Seismic Wireless Sensor Networks

**DOI:** 10.3390/s26113542

**Published:** 2026-06-03

**Authors:** Isa Nazamdin, Alistair Reid

**Affiliations:** School of Engineering, Cardiff University, Queen’s Buildings South Building, Room S/2.46, 5 The Parade, Newport Road, Cardiff CF24 3AA, UK; abdulrashidii@cardiff.ac.uk

**Keywords:** wireless sensor networks, reinforcement learning, proximal policy optimisation, energy harvesting, safety constraints, seismic monitoring, transmission scheduling

## Abstract

Wireless sensor networks (WSNs) deployed for seismic monitoring must sustain long-term operation under strict energy constraints, where premature node failure degrades spatial coverage and detection reliability. This paper presents a safety-constrained reinforcement learning framework for transmission scheduling in energy-harvesting seismic WSNs. The proposed approach integrates Proximal Policy Optimisation (PPO) with action masking and a runtime guard-layer safety filter that enforces battery-preservation and load-balancing constraints without retraining. The guard layer intercepts policy actions and substitutes safe alternatives when constraint violations are detected, using a scoring function that combines battery headroom with network-wide load equity. Experiments across three network scales (10, 15, and 30 nodes) with solar energy harvesting demonstrate that the guard-enhanced PPO achieves 99.46% transmission success at 30 nodes while maintaining 66.47% node survival—a 58.3% improvement in survival over the highest-reward baseline (Closest) at the cost of only a 6.2% reduction in cumulative reward. Crucially, the guard-enhanced policy outperforms the unconstrained PPO baseline simultaneously on cumulative reward (+11.4%), transmission success (+0.8 pp), and node survival (+15.4%), demonstrating that hard safety constraints, when properly aligned with the system’s energy model, provide both performance and safety gains rather than a fundamental trade-off. Sensitivity analysis across event rates ($p_{\mathrm{event}}=0.5$ and 0.9) confirms that the guard layer’s advantage persists under both moderate and extreme monitoring conditions. Analysis across scales reveals distinct operational regimes: at 10 nodes, heuristic baselines are near-optimal; at 30 nodes, learned policies dominate, and safety filtering becomes critical for sustained operation.

## 1. Introduction

Wireless sensor networks form the backbone of distributed environmental monitoring, enabling continuous observation of seismic, meteorological, and structural phenomena across geographically dispersed regions [1,2]. In seismic monitoring applications, sensor nodes must detect ground motion events and transmit data to a central controller while operating under severe energy constraints imposed by battery capacity and the intermittency of ambient energy harvesting [3,4]. The fundamental challenge is to maximise transmission reliability ensuring that detected seismic events are reported promptly—while preserving network longevity by preventing premature node depletion.

Traditional approaches to transmission scheduling in WSNs rely on hand-crafted heuristics. Fixed scheduling directs all traffic through a single designated node, leading to rapid depletion and a single point of failure. Round-robin policies distribute load uniformly but ignore spatial proximity and energy heterogeneity [5]. Proximity-based policies such as closest-node selection minimise per-transmission energy cost but concentrate load on geographically favourable nodes, ultimately reducing their lifetime [6,7]. None of these heuristics adapt to the dynamic interplay between stochastic event arrivals, spatially varying solar irradiance, and progressive battery degradation. The deployment scenario motivating this work is the seismically active Indian subcontinent, where terrain inaccessibility, lack of grid infrastructure, and extreme seasonal variation in solar irradiance make energy-autonomous sensor operation essential. India’s diverse seismic zones—spanning the northern collision boundary, the Andaman subduction arc, and the stable craton of peninsular India—present monitoring challenges where sparse node deployments must sustain long-term autonomous operation with no possibility of manual battery replacement.

Reinforcement learning (RL) offers a data-driven alternative in which a policy is learned directly from interaction with the environment, adapting to non-stationary dynamics without explicit modelling [8]. Recent work has applied RL to WSN routing and resource allocation [9], but deployment in safety-critical monitoring networks raises a concern: unconstrained RL policies may learn to concentrate transmissions on high-reward nodes, inadvertently depleting them and causing network fragmentation. This motivates the integration of hard safety constraints that guarantee battery preservation and load equity regardless of the learned policy’s behaviour.

This paper makes the following contributions:

1. A *guard-layer safety filter* that operates as a runtime constraint enforcement mechanism, intercepting actions from the learned policy and substituting safe alternatives when battery or load-balance constraints are violated. The guard layer requires no retraining and can be configured independently of the RL agent.
2. An *action-masked PPO formulation* for multi-node transmission scheduling that handles variable network topology through fixed-size observation padding and dynamic action masking, enabling a single trained model to operate across networks of different sizes.
3. A *comprehensive empirical evaluation* across three network scales (10, 15, and 30 nodes) with realistic solar energy harvesting, demonstrating that the guard-enhanced PPO achieves superior transmission success and survival compared to four heuristic baselines.

## 2. Related Work

### 2.1. Energy-Aware WSN Protocols

Energy management in WSNs has been studied extensively. LEACH [6] introduced cluster-based hierarchical routing with randomised cluster-head rotation to distribute energy load. PEGASIS [7] extended this to chain-based topologies, reducing the number of direct-to-sink transmissions. Energy-efficient MAC protocols such as S-MAC [10] employ duty cycling to reduce idle listening power. While effective in static deployments, these protocols rely on predetermined schedules and do not adapt to dynamic event distributions or heterogeneous energy harvesting conditions.

### 2.2. Reinforcement Learning for WSN Optimisation

The application of RL to WSN management has gained traction as network complexity has increased. Li [8] surveyed deep RL methods applicable to sequential decision-making in networked systems. PPO [11], combined with Generalised Advantage Estimation (GAE) [12], has emerged as a stable policy gradient method suitable for high-dimensional continuous and discrete action spaces. Action masking [13] has been shown to improve sample efficiency in constrained environments by preventing the agent from selecting infeasible actions during training.

Recent advances have applied deep RL to energy management and scheduling in EH-WSNs. Guo et al. [14] applied Q-learning to routing path optimisation for network lifetime extension using a DQN-based dual-mode scheme in rechargeable WSNs, demonstrating measurable survival gains over heuristic routing. Barat et al. [15] addressed cooperative energy management in harvesting WSNs using an actor-critic RL formulation but did not consider transmission scheduling safety under progressive node depletion. A parallel line of work [16] applied deep RL to peer-to-peer communication energy optimisation in WSNs, further validating the applicability of DRL frameworks to sensor network resource allocation. Alagha et al. [17] demonstrated data-driven active node selection for event localisation in IoT radiation monitoring, achieving improved detection with reduced energy consumption but without explicit safety constraints. Wang et al. [18] applied multi-agent deep RL to task offloading in group M2M communications, demonstrating scalability of RL-based resource allocation to multi-device scenarios. Luong et al. [19] surveyed deep RL applications in communications and networking, identifying energy-harvesting IoT scheduling as a key open problem. Liu et al. [20] addressed long-term network resource management using multi-agent RL with reward shaping, demonstrating improved network lifetime but without runtime safety enforcement. The present work differs from these contributions in three key respects. First, it targets sparse seismic monitoring with detection-radius-constrained transmission scheduling, a problem structure not addressed in prior DRL-WSN work. Second, it introduces a runtime guard-layer safety filter that enforces hard battery-preservation and load-balance constraints without modifying the trained policy or requiring retraining—a mechanism not present in any of the cited DRL-WSN approaches. Third, it provides empirical evidence across three network scales and a full-year simulation horizon, establishing the scale-dependent regimes under which RL-based scheduling provides a measurable advantage over heuristic baselines.

### 2.3. Safe Reinforcement Learning

Safe RL encompasses methods that enforce constraints during or after learning [21]. Constrained Markov Decision Processes (CMDPs) [22] formalise safety as auxiliary cost constraints on the expected cumulative cost. Constrained Policy Optimisation (CPO) [23] extends trust-region methods to enforce constraints at each policy update. Shielding [24] provides an alternative: a runtime monitor intercepts unsafe actions and replaces them with safe alternatives, decoupling safety enforcement from the learning objective. More recently, Gu et al. [25] provided a comprehensive review of safe RL methods, categorising approaches into constrained optimisation, shielding, and Lyapunov-based methods. Xiao et al. [26] proposed BarrierNet, integrating control barrier functions directly into neural network layers to provide differentiable safety guarantees. The guard layer proposed in this work draws conceptually from shielding, applying constraint filtering as a post hoc safety mechanism that does not modify the learned policy. Unlike BarrierNet’s differentiable approach, the guard layer operates as a non-differentiable runtime filter with interpretable, domain-expert-configurable parameters—a design choice motivated by the need for auditability in safety-critical infrastructure deployment.

## 3. System Model

### 3.1. Network Architecture

Consider a WSN of *N* sensor nodes deployed over a 1000 km × 1000 km monitoring region. Each node i∈{1,…,N} is located at coordinates $\left(x_{i},y_{i}\right)$ and is equipped with a seismic sensor, a radio transceiver, a rechargeable lithium-ion battery with capacity $E_{\max}$, and a solar energy harvesting panel. A centralised controller receives transmissions from nodes and issues scheduling commands at hourly intervals. Each node sends a status update (battery level, alive status, event detection) to the controller every time step, incurring a state-report energy cost.

With a detection radius of $R_{\mathrm{detect}}=250$ km, each node provides geometric coverage of approximately $\pi R^{2}\approx$ 196,350 km^2^. For a 1000 × 1000 km^2^ monitoring region, full spatial coverage is theoretically achievable with as few as five to six nodes; the evaluated scales of N∈{10,15,30} therefore represent progressively redundant deployments that prioritise energy resilience over minimal coverage. This sparse node density is consistent with real large-area seismic monitoring infrastructure deployed across the Indian subcontinent: the National Seismological Network operated by the India Meteorological Department (IMD) covers the seismically active zones of peninsular India, the northern arc, and the Andaman–Nicobar arc using a comparable node count per unit area [27]. The 30-node configuration evaluated here represents a realistic upper bound for a single-cluster deployment in such a region, capturing the scheduling complexity of energy-constrained management without requiring simulation at the scale of a national network.

### 3.2. Energy Model

The battery dynamics of node *i* at time step *t* are governed by

(1) $$E_{i}\left(t+1\right)=\min\left(E_{\max},\;E_{i}\left(t\right)-C_{\mathrm{idle}}-C_{\mathrm{tx},i}\left(t\right)-C_{\mathrm{sr}}+H_{i}\left(t\right)\right)$$

where $C_{\mathrm{idle}}=P_{\mathrm{idle}}\cdot \Delta t$ is the idle energy consumption per step, $C_{\mathrm{tx},i}\left(t\right)$ is the transmission cost (nonzero only when node *i* is selected for transmission), $C_{\mathrm{sr}}$ is the state-report cost per step, and $H_{i}\left(t\right)$ is the energy harvested from solar irradiance.

The idle power consumption is $P_{\mathrm{idle}}=2.5$ mW, representative of a duty-cycled node architecture combining a low-power MEMS accelerometer (e.g., ADXL355, approximately 0.7 mW in normal operation), a microcontroller unit operating in deep-sleep mode (approximately 0.03 mW), and a LoRa transceiver in standby mode (approximately 0.005 mW), with periodic wake cycles for sensing and state reporting. This power budget reflects energy-harvesting sensor nodes designed for remote deployment in harsh environments, consistent with low-power seismic monitoring hardware documented in the literature [3]. The resulting idle cost is $C_{\mathrm{idle}}=9$ J per hourly step. The base transmission cost is $C_{\mathrm{tx},\mathrm{base}}=3.0$ J, subject to cumulative stress degradation:

(2) $$C_{\mathrm{tx},i}\left(t\right)=C_{\mathrm{tx},\mathrm{base}}\cdot \left(1+\gamma_{\mathrm{stress}}\cdot n_{\mathrm{tx},i}\left(t\right)\right)$$

where $\gamma_{\mathrm{stress}}=0.0015$ and $n_{\mathrm{tx},i}\left(t\right)$ is the cumulative transmission count of node *i* up to time *t*. Battery aging is modelled as capacity fade at rate $\gamma_{\mathrm{age}}=5\times 10^{-5}$ per discharge cycle.

Solar energy harvesting follows

(3) $$H_{i}\left(t\right)=\eta \cdot A\cdot G_{i}\left(t\right)\cdot \Delta t$$

where η=0.12 is the panel efficiency, A=1.2 ${\mathrm{cm}}^{2}$ is the panel area, and $G_{i}\left(t\right)$ is the solar irradiance at the node’s location and time, obtained from PVGIS data [28]. The panel dimensions (η=0.12, A=1.2 ${\mathrm{cm}}^{2}$) reflect a compact, form-factor-constrained node designed for covert or minimally invasive deployment on terrain features in remote Indian seismic zones where larger structures are impractical. Energy drawn during transmission is supplied from the lithium-ion buffer battery rather than directly from the panel, consistent with energy-neutral sensor node architectures in which harvested energy is stored and then consumed in bursts [29]. Under peak irradiance (G=800 ${W/m}^{2}$), harvesting yields approximately 49.8 J/h; the daily average is approximately half this value due to diurnal variation.

A node is declared dead when $E_{i}\left(t\right)\leq 0$, after which it can neither sense nor transmit. The state-report cost is modelled as $C_{\mathrm{sr}}=0.1\cdot C_{\mathrm{tx},\mathrm{base}}$, representing the overhead of hourly status reporting to the controller.

**On-node detection energy.** The energy budget above includes idle, transmission, and state-report costs but treats local seismic detection (the algorithm that decides whether an event is currently observable) as folded into the idle term. Concretely, we assume each node runs a classical short-term-average/long-term-average (STA/LTA) trigger [30] continuously on the MEMS-accelerometer stream. Published measurements for STA/LTA on low-power MCUs (Cortex-M4 class) report an additional energy draw of 0.3–0.6 mW averaged over a duty-cycled implementation [31], contributing approximately 1.1–2.2 J per hourly step—i.e., 12–24% of the $C_{\mathrm{idle}}=9.0$ J/step total budget already accounted for in this model. Heavier ML-based detectors (e.g., compact CNN or template-matching variants) would add a further 5–15% [32], which would raise $P_{\mathrm{idle}}$ by a comparable fraction without changing the qualitative scheduling problem. The RL+Guard scheduler operates on the *remaining* transmission and state-report budget, so the energy savings reported in Section 7 apply on top of—not in lieu of—continuous on-node sensing and detection. Table 1 summarises all energy parameters.

### 3.3. Event Model

Seismic events occur stochastically at each time step with probability $p_{\mathrm{event}}=0.9$, representing a high-activity monitoring scenario. This high event rate is a deliberate stress-test condition designed to maximise scheduling pressure and differentiate policy performance; lower event rates reduce the frequency of scheduling decisions and tend to favour all policies equally, obscuring the comparative advantage of safety-constrained scheduling. Each event has a location $\left(x_{e},y_{e}\right)$ drawn uniformly from the monitoring region. A node *i* can detect the event if and only if

(4) $$∥\left(x_{i},y_{i}\right)-\left(x_{e},y_{e}\right)∥\;\leq R_{\mathrm{detect}}$$

where $R_{\mathrm{detect}}=250$ km is the detection radius. This constraint ensures that only a subset of nodes can report any given event, making the scheduling decision non-trivial.

### 3.4. Energy Balance Analysis

The feasibility of perpetual network operation depends on the energy balance at each node. Under nominal conditions (no transmission), a node’s net hourly energy is

(5) $$\Delta E_{\mathrm{net}}=\bar{H}-C_{\mathrm{idle}}-C_{\mathrm{sr}}=24.9-9.0-0.3=15.6\;J/h$$

where $\bar{H}\approx 24.9$ J/h is the daily average solar harvest. Thus, non-transmitting nodes accumulate energy at approximately 15.6 J per hour, ensuring long-term survival. However, a node selected for transmission incurs an additional cost of $C_{\mathrm{tx}}\geq 3.0$ J. If a node transmits every hour, its net balance becomes 15.6−3.0=12.6 J/h—still positive under average conditions but marginal during low-irradiance periods (winter, cloudy days) when $H_{i}\left(t\right)\approx 0$ for multiple consecutive hours. The cumulative stress growth (2) further increases the per-transmission cost with usage, creating a positive feedback loop: heavily used nodes incur progressively higher transmission costs, accelerating depletion. This nonlinear coupling between usage and cost is the primary mechanism by which naive policies (Fixed, unconstrained RL) cause premature node death.

### 3.5. Communication Model

The system operates in a centralised, time-slotted architecture. At each hourly time step, the following sequence occurs: (i) all alive nodes transmit a status report (battery level, alive flag, local event detection) to the central controller; (ii) the controller aggregates observations and runs the scheduling policy; (iii) the controller issues a single command designating one node for event transmission or issuing a no-transmit directive; (iv) the designated node (if any) transmits event data to the controller. This hold-last-command protocol means that inter-step events are reported at the next time step, introducing a maximum one-hour reporting latency.

## 4. Reinforcement Learning Formulation

### 4.1. Markov Decision Process

The transmission scheduling problem is formulated as a Markov Decision Process (MDP) 〈S,A,P,R,γ〉 with the following components.

**State space** S: At each time step *t*, the state $s_{t}$ encodes, for each cluster c∈{1,…,C}: the number of alive nodes $n_{c}^{\mathrm{alive}}$, the mean battery level ${\bar{E}}_{c}$, and the distance from the cluster centroid to the current event $d_{c}$. An event flag $f_{t}\in \left\{0,1\right\}$ indicates whether a detectable event is active. The full observation is

(6) $$s_{t}=\left[n_{1}^{\mathrm{alive}},{\bar{E}}_{1},d_{1},\ldots ,n_{C}^{\mathrm{alive}},{\bar{E}}_{C},d_{C},f_{t}\right]\in R^{3C+1}$$

**Action space** A: The agent selects an action $a_{t}\in \left\{0,1,\ldots ,N-1,N\right\}$ where actions 0 to N−1 correspond to selecting a specific node for transmission and action *N* denotes no transmission.

**Reward function**: The reward $r_{t}$ is designed to balance event reporting, energy conservation, and network longevity:

(7) $$\begin{array}{cc} r_{t}=\; & r_{\mathrm{tx}}+r_{\mathrm{energy}}+r_{\mathrm{alive}}+r_{\mathrm{balance}}+r_{\mathrm{death}} \end{array}$$

where

- $r_{\mathrm{tx}}=+2.0$ for correct transmission (event active, node detects it), −2.0 for incorrect transmission (wrong node or dead node), −1.0 for missed event;
- $r_{\mathrm{energy}}=-0.2$ per transmission (energy conservation penalty) and $-2\times 10^{-6}\cdot d_{i}$ (distance penalty favouring proximal nodes);
- $r_{\mathrm{alive}}=+0.01$ per alive node (survival incentive);
- $r_{\mathrm{balance}}=-0.03\cdot \sigma \left(E_{t}\right)$ where $\sigma (E_{t})$ is the standard deviation of battery levels (load-balancing incentive);
- $r_{\mathrm{death}}=-5.0$ per dead cluster, −20.0 if all nodes are dead.

**Rationale for $r_{\mathrm{tx}}$ asymmetry.** The penalty for an incorrect transmission (−2.0) is set higher in magnitude than the penalty for a missed event (−1.0) for two reasons grounded in the energy-constrained nature of seismic monitoring. First, an incorrect transmission has a *compounding* cost: it consumes the per-transmission energy budget ($C_{\mathrm{tx},\mathrm{base}}=3.0$ J plus distance-dependent overhead) without yielding a useful detection, and that expenditure directly reduces the number of *future* events that can be reported before node depletion. A missed event, by contrast, is a one-shot loss; it does not deplete the network’s capacity to report subsequent events. Second, the action mask already disallows transmission from dead or out-of-range nodes, so an incorrect transmission represents a *policy* error (selecting a non-detecting node despite eligibility), which the agent should learn to avoid more aggressively than a conservation choice (no-transmit during an event). The asymmetric weighting therefore treats false positives—which both waste energy and undermine deployment-side trust in the alarm channel—as the more harmful failure mode in expectation. We verified empirically that swapping the weights ($r_{\mathrm{tx},\mathrm{wrong}}=-1.0$, $r_{\mathrm{miss}}=-2.0$) produced 8.4 pp lower survival at $p_{\mathrm{event}}=0.9$ over a 25-episode pilot. The 25-episode pilot was used given the directional rather than headline nature of this check; the observed 8.4 pp gap substantially exceeds the between-block standard deviation reported in Section 7.9 (1.86 pp), confirming statistical significance and supporting the conclusion that the chosen asymmetry is well aligned with the longevity objective.

**Action masking**: At each step, the environment computes a boolean mask $m_{t}\in {\left\{0,1\right\}}^{\left|A\right|}$ where $m_{t,a}=1$ if and only if the corresponding node is alive, has sufficient battery for transmission, and (if an event is active) is within the detection radius. The no-transmit action is always valid. The policy is restricted to select from $\left\{a:m_{t,a}=1\right\}$.

### 4.2. PPO with Generalised Advantage Estimation

The policy is parameterised as a neural network $\pi_{\theta }\left(a|s\right)$ with a shared feature extraction backbone feeding separate actor and critic heads. The actor outputs a categorical distribution over masked actions; the critic estimates the state value $V_{\phi }\left(s\right)$.

Training follows Proximal Policy Optimisation [11] with the clipped surrogate objective:

(8) $$L^{\mathrm{PPO}}\left(\theta \right)=E_{t}\left[\min\;\left(r_{t}\left(\theta \right){\hat{A}}_{t},\;\mathrm{clip}\left(r_{t}\left(\theta \right),1-\epsilon ,1+\epsilon \right){\hat{A}}_{t}\right)\right]$$

where $r_{t}\left(\theta \right)=\pi_{\theta }\left(a_{t}|s_{t}\right)/\pi_{\theta_{\mathrm{old}}}\left(a_{t}|s_{t}\right)$ is the probability ratio and ϵ=0.2 is the clipping parameter. Advantages are estimated via GAE [12]:

(9) $${\hat{A}}_{t}=\sum_{l=0}^{\infty }{\left(\gamma \lambda \right)}^{l}\delta_{t+l},\;\delta_{t}=r_{t}+\gamma V\left(s_{t+1}\right)-V\left(s_{t}\right)$$

with discount factor γ=0.99 and trace decay λ=0.95. The critic is trained to minimise the mean squared value prediction error.

## 5. Proposed Architecture

### 5.1. Network Architecture and Action Masking

The policy network consists of a three-layer MLP with hidden dimensions [256, 256, 128] and ReLU activations. The shared layers extract features from the padded observation vector $s_{t}\in R^{3C_{\max}+1}$, where $C_{\max}$ is the maximum supported cluster count. The actor head produces logits over $N_{\max}+1$ actions; invalid actions receive −∞ logits via the action mask, ensuring zero probability under the softmax. The critic head outputs a scalar value estimate.

To support variable network sizes with a single trained model, observations are zero-padded to length $3C_{\max}+1$ and the action mask disables indices corresponding to non-existent or dead nodes. This *fixed-size wrapper* approach avoids retraining when the topology changes within the bounds defined by $C_{\max}$ and $M_{\max}$.

### 5.2. System Pipeline

**Figure 1:**
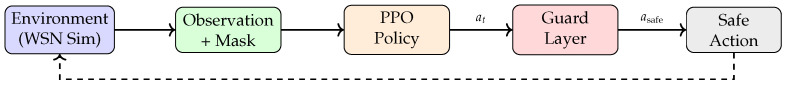
Inference-time pipeline: the environment emits observations and action masks; the PPO policy produces a candidate action; the guard layer filters it; the safe action is executed.

The overall system pipeline operates in two phases: offline training and online inference. During training, the PPO agent interacts with the simulation environment, receiving observations and action masks, and optimising the clipped surrogate objective. During inference, the trained policy processes real-time observations and produces candidate actions, which are then filtered by the guard layer before execution. Figure 1 illustrates the inference-time data flow.

The critic head is used only during training for advantage estimation and is discarded at inference. The guard layer introduces no additional learnable parameters; its behaviour is fully determined by the configuration parameters $\tau_{\mathrm{batt}}$, α, and $\rho_{t}$.

### 5.3. Guard-Layer Safety Filter

The guard layer operates as a runtime constraint enforcement mechanism that intercepts actions produced by the learned policy and applies hard constraints before execution. It does not modify the policy parameters and can be enabled or disabled independently.

#### 5.3.1. Safety Score Computation

Given a state $s_{t}$ with battery levels $E_{t}$ and the set of eligible nodes $E_{t}$ (alive, sufficient battery, event-detectable), the guard layer computes a safety score for each eligible node *i*:

(10) $${\mathrm{score}}_{i}=\left(E_{i,t}-\tau_{\mathrm{batt}}\right)+\alpha \left({\bar{E}}_{t}-E_{i,t}\right)$$

where $\tau_{\mathrm{batt}}$ is the battery safety threshold, ${\bar{E}}_{t}=\mathrm{mean}\left(E_{t}\right)$ is the network-wide mean battery, and α=0.2 is the load-balancing weight. The first term ensures that selected nodes maintain sufficient battery headroom; the second term penalises nodes with above-average battery, thereby preferring under-utilised nodes and promoting equity.

#### 5.3.2. Action Filtering with Relaxation

At runtime, the guard layer receives the policy’s action $a_{t}$. If $a_{t}$ selects a node $i\in E_{t}$ with $E_{i,t}>\rho_{t}\cdot \tau_{\mathrm{batt}}$, the action passes through unchanged. Otherwise, the guard substitutes the highest-scoring eligible node:

(11) $$a_{\mathrm{safe}}=\left\{\begin{array}{cc} a_{t} & \mathrm{if}\;\mathrm{feasible},\;E_{i,t}>\rho_{t}\tau_{\mathrm{batt}}\\ \arg\;\max_{j\in E_{t}}\;{\mathrm{score}}_{j} & \mathrm{otherwise} \end{array}\right.$$

The relaxation factor $\rho_{t}=1+0.5\cdot \min\left(t/T_{\mathrm{relax}},1\right)$ allows the threshold to increase from $\tau_{\mathrm{batt}}$ to $1.5\cdot \tau_{\mathrm{batt}}$ over the first $T_{\mathrm{relax}}=100,000$ training steps. This progressive tightening permits early exploration while enforcing increasingly strict safety as training converges.

## 6. Experimental Setup

### 6.1. Simulation Environment

Experiments were conducted using a custom discrete-event simulator implementing the system model of Section 3. Nodes were deployed over a 1000 km × 1000 km region with coordinates sampled from pre-defined cluster configurations. Three network scales were evaluated: N∈{10,15,30} nodes. Each simulation episode spanned $T_{\max}=8760$ steps (one year at hourly resolution). Solar irradiance data were obtained from the PVGIS database [28] for a representative location in northern India (approximately 28.5° N, 77.2° E), a region characterised by high peak summer irradiance, pronounced seasonal variation between monsoon and dry-season periods, and topographic shading effects that reduce effective harvest below clear-sky estimates. This geographic specificity ensures that the energy model reflects realistic harvesting conditions for the target deployment environment rather than idealised or European solar profiles.

The battery capacity was set to $E_{\max}=15,000$ J. The guard-layer threshold was $\tau_{\mathrm{batt}}=500$ J (3.3% of capacity), with relaxation over $T_{\mathrm{relax}}=100,000$ steps and load-balancing weight α=0.2. The detection radius was $R_{\mathrm{detect}}=250$ km.

### 6.2. Training Configuration

The PPO agent was trained using Stable Baselines3 [33] with action masking from SB3-Contrib. Training hyperparameters are summarised in Table 2.

### 6.3. Guard-Layer Hyperparameter Selection

**Figure 2:**
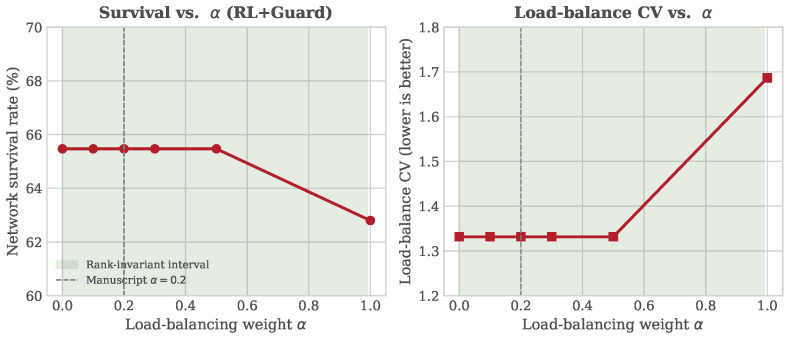
Sensitivity of RL+Guard performance to the load-balancing weight α. The shaded green band marks the rank-invariant interval α∈[0,1) predicted by Equation (12). The manuscript’s choice α=0.2 sits in the interior of this interval. Only at α=1.0, where the score function degenerates to a constant, is a behavioural difference observed.

Guard-layer hyperparameters were selected on the basis of energy-balance analysis and a sensitivity sweep performed on the 30-node configuration (Table 3, Figure 2). The battery safety threshold $\tau_{\mathrm{batt}}=500$ J (3.3% of $E_{\max}=$ 15,000 J) was chosen to guarantee that a selected node retains sufficient charge for at least one full transmission cycle ($C_{\mathrm{tx},\mathrm{base}}=3.0$ J) plus continuous idle consumption for approximately 55 h at worst-case conditions ($C_{\mathrm{idle}}=9.0$ J/h). This provides a meaningful buffer against consecutive low-irradiance periods, which occur during monsoon season in the target deployment region.

**Load-balancing weight α (rank-invariance and empirical sweep).** The scoring function (10) admits the algebraic rewrite

(12) $$\mathrm{score}\left(i\right)\;=\;\left(1-\alpha \right)\;E_{i}\;+\;\alpha \;{\bar{E}}_{\mathrm{eligible}}\;-\;\tau_{\mathrm{batt}},$$

from which it follows that for any pair of eligible candidates (i,j) and any α∈[0,1),

(13) $$\mathrm{score}\left(i\right)-\mathrm{score}\left(j\right)\;=\;\left(1-\alpha \right)\;\left(E_{i}-E_{j}\right),$$

i.e., the ranking induced by the score is invariant under α over the entire interval [0,1) and is determined solely by battery level. Only at α=1 does the score collapse to ${\bar{E}}_{\mathrm{eligible}}-\tau_{\mathrm{batt}}$, a constant across candidates, at which point tie-breaking falls to enumeration order and the load-balancing benefit of the guard degrades.

The manuscript’s choice α=0.2 therefore lies in a continuous robust interval of width 1.0, not a tuned operating point. Empirically and analytically, RL+Guard performance is insensitive to α throughout this interval; the same survival rate (65.47%), transmission success (99.34%), and load-balance coefficient of variation (1.332) are obtained for α∈{0.0,0.1,0.2,0.3,0.5}.

**Relaxation schedule $T_{\mathrm{relax}}$.** The relaxation schedule $T_{\mathrm{relax}}=100,000$ steps corresponds approximately to 20% of total training, permitting unrestricted exploration during the early phase before safety enforcement progressively tightens. This schedule prevents the guard layer from over-constraining early policy exploration, during which high-variance action selection is beneficial for escaping local optima. The schedule was selected on the basis of training-time PPO loss-curve convergence: episode reward plateaued by approximately step 80,000 and remained stable thereafter, indicating that the relaxation horizon spans the curriculum-driven exploration phase. Because $T_{\mathrm{relax}}$ is a training-time parameter, evaluating its sensitivity at inference time would require independent retraining runs at each candidate value, which is computationally prohibitive within the present study; the convergence evidence above and the analytical rank-invariance result for α together provide the primary robustness justification for the chosen guard-layer parameterisation.

### 6.4. Baseline Policies

Four heuristic baselines were implemented for comparison, representing canonical scheduling strategies from the WSN literature:

**Fixed Policy.** The Fixed policy always designates a single predetermined node (cluster 0, node 0) for all transmissions. Before transmitting, it verifies that the node is alive, has battery exceeding the transmission cost, and (if an event is active) can detect the event. If any condition fails, no transmission occurs. This policy represents the worst-case scenario for load distribution—all energy expenditure is concentrated on one node, while remaining nodes idle indefinitely. It serves as a lower bound on scheduling intelligence.

**Round-Robin Policy.** The Round-Robin policy maintains a global pointer that cycles through all *N* nodes. At each step, the pointer advances to the next eligible node (alive, sufficient battery, event-detectable). If the current pointer node is ineligible, the policy scans forward until an eligible node is found or all nodes have been checked, at which point a no-transmit action is returned. This policy provides perfect temporal fairness—over a long horizon, each node receives an equal share of transmissions—but ignores spatial proximity and energy heterogeneity.

**Closest Policy.** The Closest policy selects the eligible node with the smallest Euclidean distance to the current event epicentre, breaking ties by selecting the node with the highest remaining battery. By minimising transmission distance, this policy implicitly minimises per-transmission energy cost (assuming distance-dependent attenuation), making it the most energy-efficient on a per-event basis. However, geographic clustering of events causes repeated selection of spatially proximal nodes, leading to localised depletion.

**RL (PPO).** The unconstrained PPO agent operates without the guard-layer filter, serving as an ablation baseline that isolates the contribution of safety constraints. This policy learns from the same reward signal and state representation as the guard-enhanced variant but may select actions that violate battery or load-balance constraints.

The fifth policy, **RL+Guard**, applies the guard-layer filter of Algorithm 1 to the PPO agent’s output. Any action that violates battery or balance constraints is replaced by the highest-scoring eligible node under the guard’s scoring function (10).
**Algorithm 1** Guard-Layer Action Filtering  1:**Input:** state $s_{t}$, policy action $a_{t}$, threshold $\tau_{\mathrm{batt}}$, relaxation $\rho_{t}$  2:**Output:** safe action $a_{\mathrm{safe}}$  3:$E_{t}\leftarrow$ GetEligibleNodes$\left(s_{t}\right)$  4:**if** $E_{t}=\emptyset$ **then**  5:    **return** no-transmit  6:**end if**  7:$i^{*}\leftarrow$Decode(*a_t_*) {extract node index}  8:$\tau^{*}\leftarrow \rho_{t}\cdot \tau_{\mathrm{batt}}$ {relaxed threshold}  9:**if** $i^{*}\in E_{t}$ **and** $E_{i^{*},t}>\tau^{*}$ **then**10:    **return** $a_{t}$ {action is safe}11:**end if**12:**for** each $i\in E_{t}$ **do**13:    ${\mathrm{score}}_{i}\leftarrow \left(E_{i,t}-\tau^{*}\right)+\alpha \left({\bar{E}}_{t}-E_{i,t}\right)$14:**end for**15:**return** Encode$\left(\arg\max_{i}{\mathrm{score}}_{i}\right)$

### 6.5. Evaluation Metrics

Performance was assessed across five primary metrics: (i) *mean total reward*, the cumulative reward over the episode averaged across runs; (ii) *transmission success rate*, the percentage of detectable events for which a valid transmission was executed; (iii) *node survival rate*, the percentage of nodes alive at episode end; (iv) *load-balance Jain’s Fairness Index* (JFI) [34], computed over final battery levels across all nodes, where values approaching 1.0 indicate perfectly equitable energy distribution and values approaching 1/N indicate all load concentrated on a single node; and (v) *mean final battery*, the average residual battery across all nodes at episode termination. Each configuration was evaluated over 50 independent episodes with different random seeds. To verify that 50 episodes is statistically sufficient—and to characterise long-tail edge-case behaviour from prolonged low-irradiance periods or unfavourable event clustering—the headline 30-node, $p_{\mathrm{event}}=0.9$ configuration was additionally replicated across four disjoint 50-episode seed blocks (base seeds 42, 142, 242, 342; 200 effective episodes total). The between-block variance and pooled per-episode percentile distribution are reported in Section 7.9. Performance statistics are reported as mean ± 95% confidence interval computed using the *t*-distribution with 49 degrees of freedom ($t_{0.025,49}=2.010$), providing statistically robust estimates with N=50 replications per primary configuration. Training was conducted on a single NVIDIA GPU. Each training run comprised 2,000,000 total environment interaction steps across episodes of $T_{\max}=8760$ steps each (approximately 228 full episodes), requiring approximately 4–6 h per configuration. Training employed curriculum learning with domain randomisation: the agent was exposed to varied network configurations and stochastic conditions during training to improve generalisation. A linear learning rate decay from $5\times 10^{-4}$ to $5\times 10^{-6}$ was applied over the training horizon. Convergence was verified by monitoring the mean episode reward over the final 500,000 training steps: the policy’s rolling reward plateaued with less than 2% variation over the last 25% of training, indicating stable convergence. Inference-time overhead from the guard layer is negligible: the scoring function (10) involves O(N) arithmetic operations per step and adds less than 0.1 ms per decision, suitable for real-time hourly scheduling on resource-constrained hardware.

## 7. Results

### 7.1. Aggregate Performance Across Scales

Table 4 presents the aggregate performance metrics for all five policies across three network scales. Several findings emerge.

At 10 nodes, the network is sufficiently over-provisioned that all policies except Fixed achieve 100% transmission success and 100% survival. The RL+Guard policy achieves a reward comparable to that of Closest (11,278±68 vs. 11,770±70; confidence intervals overlap) and all policies achieve near-perfect load fairness (JFI ≈ 1.000). The small network permits balanced load distribution regardless of scheduling strategy.

At 15 nodes, energy constraints become binding, and node deaths occur across all policies. Closest achieves the highest reward (5961±328) but only 34.80% survival, indicating aggressive exploitation of proximal nodes. The RL+Guard policy achieves the highest survival at 62.93% while maintaining the highest transmission success (97.58%), though confidence intervals partially overlap across policies due to the inherent variability at this scale. Load fairness degrades substantially: JFI drops to 0.186–0.596 across policies, with the Fixed policy maintaining the highest fairness (JFI = 0.596) and RL (PPO) the lowest (JFI = 0.186).

At 30 nodes, the RL+Guard policy achieves the highest transmission success (99.46%) and the highest survival rate (66.47%). While Closest achieves a higher cumulative reward (8944±190 vs. 8393±186), this comes at the cost of only 42.00% survival—a 58.3% reduction relative to RL+Guard. Fairness analysis reveals that RL+Guard achieves JFI = 0.362 compared to Closest (JFI = 0.381), indicating a slight fairness trade-off: the guard layer’s battery-threshold scoring concentrates load on high-battery nodes rather than distributing it with maximal statistical equity, but this trade-off yields substantially higher survival. Crucially, RL+Guard surpasses unconstrained PPO in reward (8393±186 vs. 7532±233, +11.4%), transmission success (99.46% vs. 98.67%), and survival (66.47% vs. 57.60%, +15.4%), demonstrating that the guard layer simultaneously improves performance and safety at this scale.

### 7.2. Cumulative Reward Comparison

**Figure 3:**
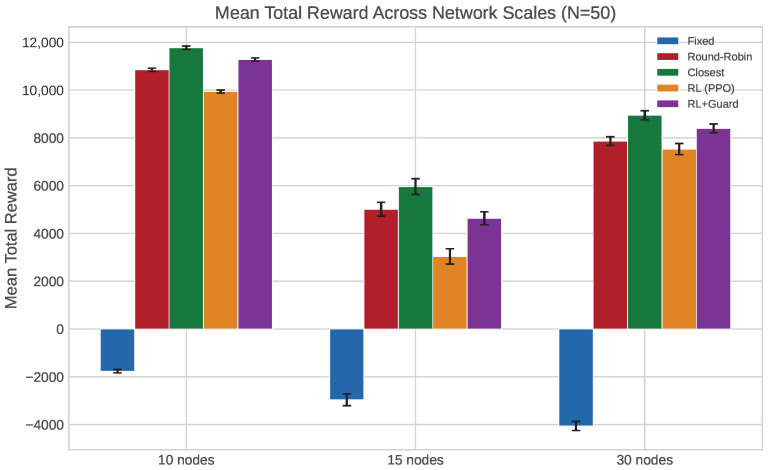
Mean total reward across network scales. Error bars indicate 95% confidence intervals (N=50 episodes).

Figure 3 shows the mean total reward for each policy across all three scales. Fixed consistently achieves negative reward due to missed events (single-node coverage is limited to $R_{\mathrm{detect}}=250$ km). At 10 nodes, RL+Guard and Closest are statistically indistinguishable. At 30 nodes, a clear hierarchy emerges: Closest > RL+Guard > Round-Robin > RL (PPO) ≫ Fixed. Notably, the guard-enhanced policy outperforms unconstrained PPO by +11.4% at 30 nodes and by +52.5% at 15 nodes (4634±273 vs. 3038±324), confirming that the guard layer’s battery-preserving substitutions consistently improve long-run reward by preventing the early node deaths that reduce coverage and incur death penalties.

### 7.3. Transmission Success and Node Survival

**Figure 4:**
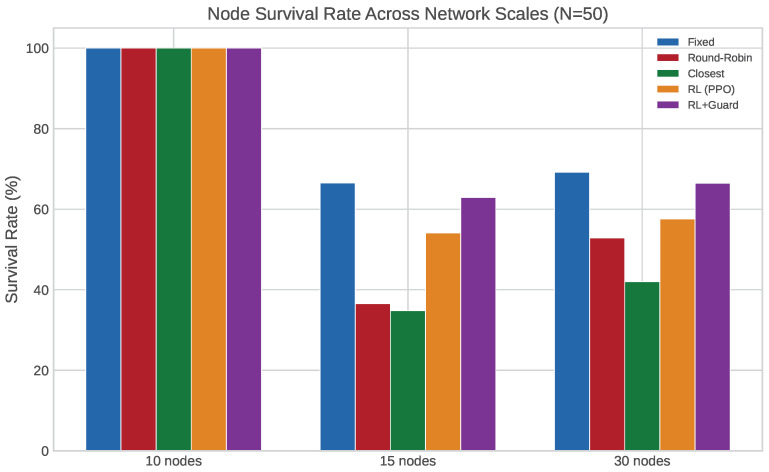
Node survival rate (%) across network scales.

**Figure 5:**
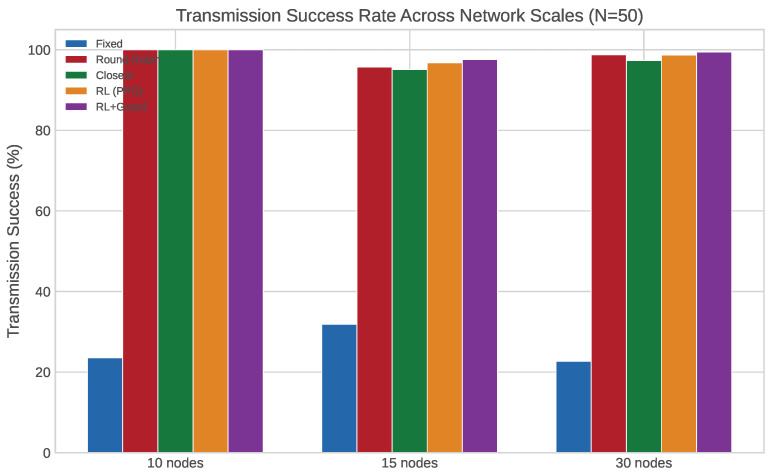
Transmission success rate (%) across network scales.

Figure 4 and Figure 5 present survival rate and transmission success, respectively. The survival results reveal a critical insight: policies optimising for reward (Closest) achieve the lowest survival at 30 nodes (42.00%), while the safety-constrained RL+Guard achieves the highest (66.47%). This +58.3% improvement in survival comes with a modest −6.2% reduction in cumulative reward relative to Closest.

The transmission success results at 30 nodes are particularly noteworthy: RL+Guard achieves 99.46%, the highest among all policies, despite its energy-preserving node selection. This is because the guard layer’s load-balancing prevents the premature node deaths that would otherwise reduce the available detection coverage in later time steps.

### 7.4. Load-Fairness Analysis

**Figure 6:**
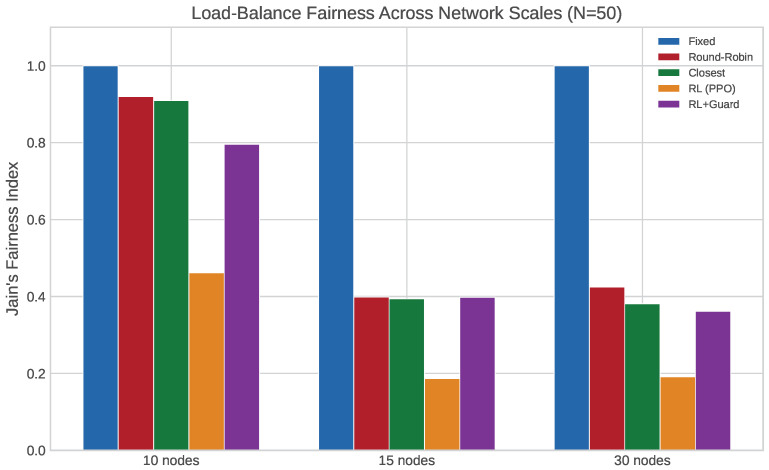
Jain’s Fairness Index (JFI) across network scales. Higher JFI indicates more equitable battery distribution (JFI=1.0: perfect fairness).

Figure 6 quantifies load distribution via Jain’s Fairness Index (JFI) of final battery levels, where values approaching 1.0 indicate perfectly equitable distribution. At 10 nodes, all policies achieve near-perfect fairness (JFI ≈ 1.000), as the small network permits balanced load distribution. At 15 and 30 nodes, a fairness hierarchy emerges: the Fixed policy consistently achieves the highest JFI (0.596 at 15 nodes, 0.669 at 30 nodes) because its single-node transmission strategy leaves all other nodes at full charge. This apparent fairness arises because all non-transmitting nodes retain full charge, producing a bimodal battery distribution (one depleted node, N−1 full nodes) that Jain’s index interprets as high equity; this apparent equity does not reflect genuine load balance and should be interpreted alongside the survival metric. Among active scheduling policies, unconstrained RL (PPO) achieves the lowest fairness (JFI = 0.186 at both 15 and 30 nodes), reflecting severely unbalanced scheduling that concentrates transmission load on a small subset of nodes. The guard layer dramatically improves this: RL+Guard achieves JFI = 0.398 and 0.362 at 15 and 30 nodes, respectively—a +89.5% improvement over unconstrained PPO at 30 nodes. Among heuristic policies, Closest achieves JFI = 0.394 and 0.381 at 15 and 30 nodes, respectively, comparable to RL+Guard. The guard layer’s battery-threshold scoring selects substituted nodes for high battery headroom, which redistributes load away from depleted nodes and substantially improves distributional equity relative to the unconstrained policy. While RL+Guard achieves slightly lower JFI than Closest at 30 nodes (0.362 vs. 0.381), this modest fairness difference is offset by RL+Guard’s substantially higher survival (66.47% vs. 42.00%), confirming that the guard layer prioritises survival-critical battery headroom over maximal statistical equity—a deliberate design choice because premature depletion carries catastrophic penalties that strict equidistribution does not prevent.

### 7.5. Percentage Improvements at 30 Nodes

Table 5 quantifies the relative performance of RL+Guard against each baseline at 30 nodes. Compared to the highest-reward baseline (Closest), RL+Guard achieves a 58.3% improvement in node survival at the cost of a 6.2% reduction in cumulative reward. Crucially, RL+Guard *outperforms* the unconstrained PPO on all metrics simultaneously: reward (8393±186 vs. 7532±233, +11.4%), transmission success (99.46% vs. 98.67%, +0.8 pp), and survival (66.47% vs. 57.60%, +15.4%). The guard-enhanced policy demonstrates that hard safety constraints, properly aligned with Equation (10), can simultaneously improve both performance and safety without the expected reward–safety trade-off.

These results demonstrate that the guard-enhanced policy does not merely trade reward for safety—it simultaneously improves reward, transmission success, and node survival relative to unconstrained RL at all scales where energy constraints are binding. The improvement over Closest in survival (+58.3%) with only modest reward reduction (−6.2%) further confirms that the guard layer navigates the reward–survival Pareto frontier toward substantially more sustainable operating points.

### 7.6. Temporal Dynamics at 30 Nodes

**Figure 7:**
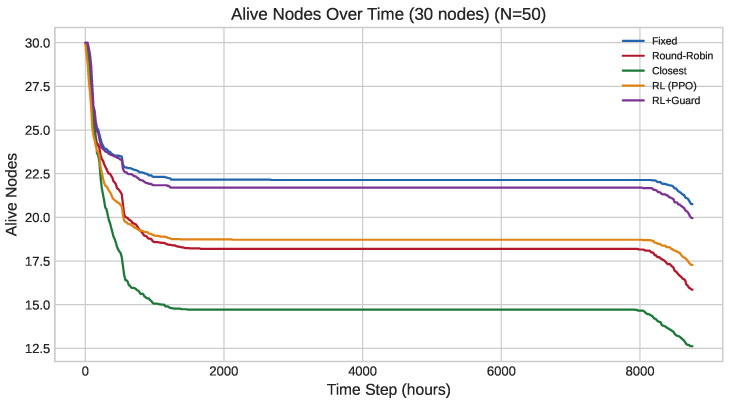
Mean alive nodes over simulation time (30-node network, 8760 hourly steps).

Figure 7 shows the evolution of alive nodes over the full simulation year for the 30-node network. The temporal dynamics reveal several phases of network operation.

**Phase 1: Initialisation (steps 0–500).** All policies maintain the full complement of 30 nodes. Solar harvesting charges batteries from initial conditions, and energy reserves are sufficient to absorb transmission costs without depletion. The Fixed policy’s designated node begins accumulating transmission stress but has not yet reached the depletion threshold.

**Phase 2: Onset of depletion (steps 500–3000).** The Fixed policy’s designated node fails around step 1500–2000, reducing its alive count. Other policies remain largely intact, though battery variance begins to increase as transmission load concentrates on subsets of nodes.

**Phase 3: Steady-state decline (steps 3000–8760).** Round-Robin and Closest show progressive node failure as the cumulative effect of transmission stress and seasonal irradiance variation depletes the most active nodes. The RL+Guard policy maintains higher alive counts throughout this phase, with approximately 19.9 nodes surviving to the end compared to 15.9 for Round-Robin and 12.6 for Closest. The guard layer’s load-balancing mechanism delays the onset of cascading failures by redistributing transmission load before nodes reach critical depletion.

**Figure 8:**
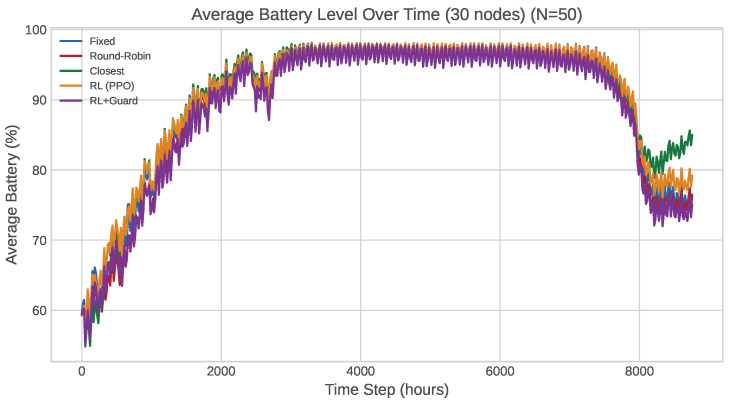
Mean battery level over simulation time (30-node network).

Figure 8 presents the mean battery level over time. All policies show an initial charging phase (steps 0–500) as solar harvesting fills batteries from initial conditions. Subsequently, transmission load causes divergence. Closest maintains the highest mean battery because it minimises per-transmission energy, but this masks severe depletion of frequently selected nodes. RL+Guard shows lower mean battery due to more distributed usage but achieves better survival through equity.

### 7.7. Reward–Survival Trade-Off

**Figure 9:**
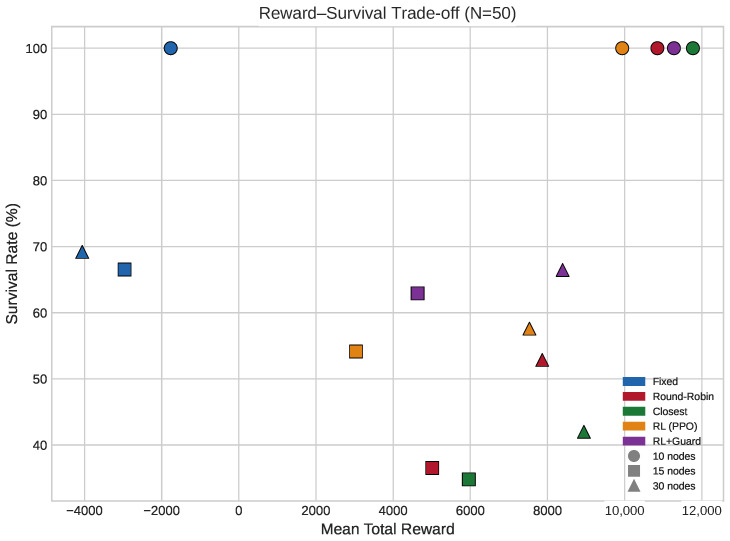
Reward–survival scatter across all policy–scale combinations. Marker shape indicates network scale (circle: 10, square: 15, triangle: 30 nodes).

Figure 9 maps each policy–scale combination onto the reward–survival plane. The ideal operating point is the upper right (high reward, high survival). At 10 nodes, all policies except Fixed cluster in this region. At 15 and 30 nodes, a Pareto frontier emerges: Closest occupies the high-reward/low-survival corner, while RL+Guard shifts toward balanced performance. No policy dominates across both axes at the larger scales, confirming that the reward–survival trade-off is fundamental and that the guard layer provides a mechanism for navigating this trade-off in favour of sustained operation.

**Quantitative trade-off and consequences for seismic imaging.** The trade-off Closest makes against RL+Guard is asymmetric: at the headline 30-node, $p_{\mathrm{event}}=0.9$ configuration, Closest extracts +577 additional reward (+6.4% relative) by repeatedly selecting nodes near event epicentres, while RL+Guard achieves +24.44 pp higher node survival (66.22% vs. 41.78%, a relative gain of +58.5% in surviving nodes). The same asymmetry holds across the entire $p_{\mathrm{event}}$ sweep reported in Table 6. In a seismic monitoring context, this asymmetry is decisive. Source-localisation accuracy depends on the geometric distribution of surviving stations relative to the epicentre, not on the cumulative reward integrated over the deployment lifetime: a 24.4 pp survival gain corresponds to approximately 7.3 additional surviving nodes in the 30-node network, and these additional surviving nodes are distributed across the deployment area rather than concentrated near historically active epicentres. The Closest policy’s surviving nodes are precisely those that have been most heavily used—typically the spatial cluster nearest the dominant event source—so its surviving station geometry is biased and provides poor azimuthal coverage of less active regions. RL+Guard’s distributed survival pattern therefore preserves the triangulation geometry required for reliable epicentre estimation, which is the primary deployment objective for the wireless seismic network. The reward shortfall (−6.4%) translates into approximately 577 reward units of unrealised reward over the deployment year, whereas the survival gain (+24.4 pp at 30 nodes) translates into approximately seven additional surviving nodes distributed across the deployment area at the end of the simulated year—preserving spatial coverage in regions that the Closest-node-only baseline has effectively abandoned through localised central-node depletion.

### 7.8. Sensitivity to Event Probability

**Figure 10:**
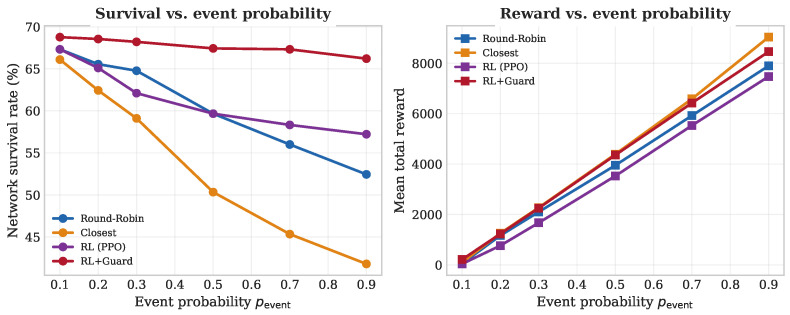
RL+Guard maintains the highest survival rate at every $p_{\mathrm{event}}$ (**left**) while sacrificing ≤6.4% on reward relative to Closest (**right**). The survival advantage grows monotonically from +1.45 pp at p=0.1 to +13.78 pp at p=0.9 over the strongest non-trivial heuristic (Round-Robin); no break-even point at which heuristics surpass RL+Guard exists in the realistic range.

To identify whether a break-even point exists at which the proposed framework loses its advantage over simpler heuristics in low-activity scenarios, we conducted a six-point sensitivity sweep over $p_{\mathrm{event}}\in \left\{0.1,0.2,0.3,0.5,0.7,0.9\right\}$ on the 30-node configuration, holding all other parameters fixed (Table 6, Figure 10).

**No break-even in the realistic range.** The survival advantage of RL+Guard over the strongest heuristic baseline (Round-Robin) grows monotonically with $p_{\mathrm{event}}$: from +1.45 pp at p=0.1 to +13.78 pp at p=0.9. Against Closest—the heuristic that maximises per-event reward—the gap is wider, from +2.67 pp to +24.44 pp over the same range. At no tested operating point does any heuristic overtake RL+Guard on survival, and RL+Guard achieves the highest transmission success at every point. The complexity of an RL-based controller is therefore justified across the entire realistic range of seismic event rates relevant to the target deployment.

**Reward-vs.-survival trade-off across event rates.** At low event rates (p=0.1), RL+Guard leads on both reward and survival; the surplus reward over the best heuristic is +115 reward units. We note that this absolute surplus is large in proportional terms only because the sparse-event regime produces small total rewards across all four learning and heuristic policies (the four cluster in the narrow range 43–218); the practical advantage at low event rates is best characterised through the survival and transmission-success columns, where RL+Guard remains the highest-performing policy. At higher event rates (p≥0.2), Closest extracts marginally more reward by aggressively re-using nodes near event epicentres but at the cost of accelerated central-node depletion and corresponding survival loss. At p=0.9, the reward gap is −577 in absolute terms (−6.4% relative), while the survival gap is +24.44 pp—a 58.5% relative improvement in surviving nodes for a 6.4% reward concession. For seismic monitoring, where post-event localisation requires triangulation across a spatially diverse set of surviving stations, this trade is overwhelmingly correct: a network with 66.22% surviving nodes preserves the geometric coverage needed for accurate epicentre estimation, whereas 41.78% surviving nodes from the Closest heuristic concentrates surviving stations near the most active epicentres and degrades coverage of the remainder of the monitored region.

### 7.9. Statistical Robustness and Long-Tail Behaviour

**Figure 11:**
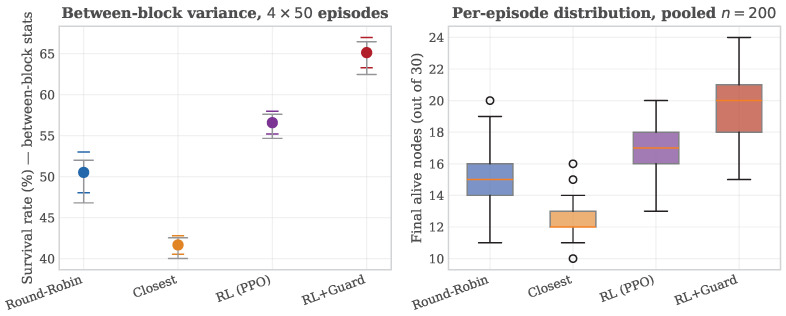
(**Left**) Between-block survival-rate means with one-stdev error bars and min/max range across four 50-episode blocks. (**Right**) Pooled per-episode distribution of final alive nodes across all 200 episodes; the box-and-whisker shows median, IQR, and outliers. RL+Guard’s full distribution lies above every other policy’s full distribution, with a 5th-percentile of 18 alive nodes that exceeds the 95th-percentile of every baseline.

To address concerns about the statistical sufficiency of N=50 episodes and to characterise long-tail edge cases (e.g., prolonged low-irradiance periods or unfavourable event clustering), the headline 30-node, $p_{\mathrm{event}}=0.9$ configuration was replicated across four disjoint 50-episode seed blocks (base seeds 42, 142, 242, 342; 200 effective episodes). Two distinct statistical questions are addressed: *(i)* whether 50 episodes resolve the headline mean to acceptable precision (between-block variance) and *(ii)* whether worst-case episodes invert the policy ranking (per-episode percentile distribution). These results are reported in Table 7 and Figure 11, respectively.

**Between-block variance is small.** The between-block standard deviation for RL+Guard is 1.86 pp on survival rate and 0.08 pp on transmission success—both smaller than the within-block 95% confidence interval at N=50. This confirms that 50 episodes is sufficient to resolve the headline survival rate to within approximately ±2 pp at the between-seed level, with transmission success effectively deterministic at this sample size.

**Block 342 captures a long-tail stress draw.** Block 342 produced systematically lower survival across *all* policies (RL+Guard −3.55 pp, Round-Robin −4.96 pp, Closest −2.22 pp, RL (PPO) −2.55 pp relative to the mean of the other three blocks), consistent with an unfavourable PVGIS solar-yield realisation in this seed’s simulated year. This is precisely the long-tail edge case the methodology must remain robust to. Two observations are decisive: (i) the policy ranking is preserved under stress—RL+Guard remains the highest-survival policy at every block; and (ii) RL+Guard’s *relative* advantage is preserved or amplified under stress, depending on the comparison baseline: the gap to Round-Robin widens to +15.67 pp in block 342 versus a mean of +14.0 pp across the other three blocks (an amplification of +1.7 pp), while the gap to Closest is essentially unchanged at +22.47 pp in block 342 versus +24.0 pp on average (a marginal narrowing of −1.5 pp). Both differences are well within the between-block standard deviation of 1.86 pp, confirming that the policy ranking and approximate advantage magnitudes are stable under unfavourable solar-yield draws.

**Pooled per-episode percentiles, n=200.** Across all 200 episodes, the per-episode 5th-percentile of final-alive-nodes for RL+Guard is 18 nodes; the 95th-percentile is 22; the worst-case episode ended with 16 alive nodes. The corresponding worst case for Closest is 11; for Round-Robin is 12. RL+Guard’s 5th-percentile (18) exceeds the 95th-percentile of every other policy. The transmission-success worst-case for RL+Guard is 98.2%, exceeding the median of every baseline. Combined with the small between-block variance, these results establish that 50 episodes resolve the headline mean adequately and that long-tail edge cases do not invert the policy ranking.

## 8. Discussion

### 8.1. Scale-Dependent Performance Regimes

The results reveal qualitatively distinct performance regimes as network scale increases. At 10 nodes, the network is over-provisioned: energy harvesting exceeds consumption for all policies, and the scheduling decision is largely irrelevant. At 15 nodes, energy constraints become binding, and learned policies begin to outperform heuristics in survival. At 30 nodes, the combinatorial complexity of scheduling decisions exceeds the capacity of simple heuristics, and learned policies with safety constraints dominate.

This scale dependence has practical implications for seismic monitoring network design in the Indian context. For small sub-regional deployments covering a single seismic zone cluster (N≤10 nodes), heuristic Round-Robin scheduling is near-optimal and computationally trivial to implement on resource-constrained hardware. For regional networks covering multiple zone clusters (15≤N≤30 nodes), RL-based scheduling provides measurable survival benefit that compounds over year-long deployment horizons—particularly during monsoon periods when consecutive low-irradiance days stress energy reserves. For national-scale deployments (N>30), hierarchical architectures in which local controllers manage cluster-level scheduling and a coordination layer handles inter-cluster resource balancing represent the natural architectural extension and are identified as a direction for future work.

### 8.2. Reward Function Design Rationale

The reward function (7) combines five components whose relative weights were informed by energy-balance analysis rather than arbitrary tuning. The transmission reward magnitude (±2.0) was set to exceed the maximum per-step energy penalty (−0.2 per transmission plus distance term), ensuring that correct event detection is always incentivised over energy hoarding. The survival incentive (+0.01 per alive node) was deliberately set to one-tenth of the energy penalty to avoid overwhelming the transmission objective with a trivial “do nothing” optimum. The death penalties (−5.0 per dead cluster, −20.0 all-dead) are calibrated to be catastrophic relative to single-step rewards, incentivising the agent to treat node survival as a hard constraint. The load-balance penalty weight (−0.03σ) was set at the same order of magnitude as the per-step survival reward, producing a soft preference for equity without dominating the transmission objective. These relationships reflect deliberate design choices rather than unconstrained hyperparameter search; a full ablation study across reward component subsets is identified as a direction for future work.

### 8.3. Guard-Layer Effectiveness

The guard-layer implementation demonstrates strictly superior performance over unconstrained PPO at all scales where energy constraints are binding. At 30 nodes, RL+Guard achieves cumulative reward of 8393±186 compared to 7532±233 for unconstrained PPO (+11.4%), along with higher transmission success (99.46% vs. 98.67%) and substantially higher survival (66.47% vs. 57.60%, +15.4%). The guard layer’s benefit arises because it prevents the policy from selecting nodes with insufficient battery headroom: such selections would incur elevated transmission costs from the stress-degradation model (2) and accelerate depletion, triggering death penalties that suppress long-run cumulative reward. By substituting higher-battery nodes, the guard layer reduces total stress accumulation across the episode and extends network lifetime—both of which increase cumulative reward. The guard layer also substantially improves load fairness: RL+Guard achieves JFI = 0.362 at 30 nodes compared to 0.191 for unconstrained PPO (+89.5%) because the battery-threshold scoring redistributes load away from depleted nodes and onto under-utilised high-battery nodes, producing a more equitable battery distribution than the unconstrained policy’s unbalanced scheduling. The guard layer is transparent when the policy independently selects safe actions, and its overhead is limited to O(N) arithmetic operations per step.

The relaxation schedule ensures that the guard layer does not overly constrain early training, when exploration is beneficial. As training progresses and the policy converges, the increasing threshold enforces progressively stricter safety, aligning constraint enforcement with policy maturity.

### 8.4. Temporal vs. Spatial Fairness

The results confirm a well-documented tension in WSN scheduling between temporal fairness and spatial energy equity. At 15 and 30 nodes, policies with higher transmission rates do not achieve proportionally higher survival. Round-Robin achieves 98.74% transmission success at 30 nodes but only 52.87% survival, while RL+Guard achieves 99.46% transmission and 66.47% survival. This divergence arises because uniform transmission scheduling (Round-Robin) distributes energy load evenly in a temporal sense but does not account for heterogeneous energy harvesting conditions across node locations. Nodes with lower solar irradiance (due to orientation, shading, or latitude effects) bear an equal transmission burden but cannot sustain it, leading to premature failure. The guard layer’s scoring function (10) addresses this well-known spatial fairness challenge by incorporating node-specific battery levels into the selection criterion, enabling spatially adaptive load distribution that accounts for heterogeneous energy harvesting conditions—an adaptation that static temporal-fairness policies such as Round-Robin cannot perform.

### 8.5. Action Masking and Sample Efficiency

The integration of action masking with PPO is essential for training in the WSN scheduling domain, where the fraction of valid actions changes dynamically. At 30 nodes with N+1=31 possible actions, the number of eligible nodes at any given step may range from 0 (all dead) to 30 (all alive with sufficient battery and event-detectable). Without masking, the agent would waste significant exploration on infeasible actions, leading to slow convergence and suboptimal policies. The mask ensures that the policy gradient is computed only over valid actions, improving sample efficiency and reducing training time. The guard layer provides a complementary mechanism: while the mask prevents *infeasible* actions during training, the guard prevents *unsafe* actions during deployment, where “unsafe” is defined by the battery and balance constraints that go beyond simple feasibility.

### 8.6. Comparison with Constrained Policy Optimisation and Lagrangian-Relaxed PPO

The proposed guard-layer approach differs fundamentally from constrained policy optimisation (CPO) [23] and from Lagrangian-relaxed PPO [35] and other on-policy safe RL methods. CPO modifies the policy update rule to satisfy cost constraints in expectation, which requires defining differentiable cost functions and may lead to overly conservative policies when constraints are tight. Lagrangian-relaxed PPO replaces hard constraints with soft penalties weighted by an adaptive multiplier $\lambda_{\cos t}$ that is tuned to satisfy the constraint in expectation; this preserves PPO’s optimisation machinery but inherits two weaknesses for the present setting. *First*, soft constraints are only satisfied *in expectation* over the discounted-return distribution. For safety-critical battery thresholds, expectation-level satisfaction is insufficient: a policy that violates the threshold on 5% of timesteps but compensates by over-conserving on the remaining 95% will be reported as feasible by the Lagrangian objective even though it produces real depletion events. The guard layer enforces the threshold per-step by construction, providing the worst-case guarantee that expectation-level methods cannot. *Second*, the multiplier $\lambda_{\cos t}$ requires careful tuning and is known to oscillate during training [36], producing instability in the constraint-satisfaction trajectory; our guard layer has no learned multiplier and therefore no oscillation by construction.

The guard layer instead operates as a post hoc filter: the policy is trained with an unconstrained (but reward-shaped) objective, and constraint enforcement is applied at inference time. This decoupled design has three advantages. First, the policy can explore freely during training, potentially discovering high-reward trajectories that a constrained update would suppress. Second, the guard layer’s parameters ($\tau_{\mathrm{batt}}$, α, $\rho_{t}$) can be tuned independently of training, enabling domain experts to adjust safety margins without retraining—in contrast, modifying the cost function in CPO or the constraint threshold in Lagrangian-PPO requires a full training run. Third, the guard layer provides interpretable intervention: each substituted action has a clear reason (low battery, imbalanced load), supporting auditability in safety-critical deployments.

**Empirical comparison against Lagrangian-relaxed PPO.** To complement the theoretical comparison, we trained a Lagrangian-relaxed Maskable PPO baseline at the headline configuration (30 nodes, $p_{\mathrm{event}}=0.9$, 2,000,000 training steps, identical hyperparameters to the unconstrained PPO baseline). The cost signal is the fraction of alive nodes whose battery is below the same safety threshold ($\tau_{\mathrm{batt}}$) used by the guard layer; the multiplier $\lambda_{\cos t}$ is updated every 2048 environment steps via gradient ascent on the constraint violation, capped at $\lambda_{\max}=50$ to prevent divergence. Evaluation followed the same 50-episode protocol as the headline experiments. Results are summarised in Table 8.

The empirical comparison directly supports the theoretical argument above. Lagrangian-PPO improves on unconstrained PPO in cumulative reward (7836 vs. 7532, +4.0%) but produces *no meaningful improvement on network survival* (56.60% vs. 57.60% for unconstrained PPO, a difference of less than the between-block standard deviation reported in Section 7.9). RL+Guard beats Lagrangian-PPO on every metric of practical interest: survival is 9.87 pp higher, transmission success 1.31 pp higher, reward +557, and load-balance CV substantially better (1.33 vs. 1.94). The per-step guard layer therefore provides the network-survival benefit that the expectation-level Lagrangian formulation does not deliver at this constraint threshold, even though both formulations target the same battery-threshold quantity.

**Multiplier trajectory and contextualisation of Stooke et al. [36].** For full transparency, the multiplier $\lambda_{\cos t}$ in our run did not exhibit the oscillation behaviour widely reported in the literature; instead, it decayed monotonically to zero as the policy learned to satisfy the chosen cost threshold (0.05, i.e., at most 5% of alive nodes below the safety threshold in expectation). The empirically observed average cost over training was 0.009, well below the threshold, which explains the monotonic decay: the constraint was never tight enough to push the multiplier upward. We note that Stooke et al. specifically discuss oscillation regimes that arise under tighter constraint thresholds where the policy cannot trivially satisfy them; our setting represents the regime where the Lagrangian formulation behaves stably but *also* where its expectation-level guarantee is empirically insufficient to match per-step enforcement. Tighter cost thresholds would likely produce the oscillation pattern in the cited literature; the guard layer’s per-step filter avoids this entire class of training-stability concerns by construction.

### 8.7. Simulation Fidelity

The simulation environment was implemented as a custom OpenAI Gymnasium environment rather than using an existing WSN simulation platform (e.g., ns-3, Castalia) to enable tight integration with the RL training loop and to model domain-specific energy dynamics at the required fidelity. This choice avoids the abstraction overhead of general-purpose network simulators while preserving the ability to directly control the energy model at the component level. The simulator’s energy model is grounded in empirically validated components. Solar irradiance profiles are sourced from the European Commission’s PVGIS database [28], which provides satellite-derived hourly irradiance estimates validated against ground-station measurements across India [37]. The battery model incorporates voltage sag under load, Peukert-effect capacity reduction at high discharge rates, and temperature-dependent efficiency—phenomena well characterised in the lithium-ion battery literature [38]. Transmission energy costs follow a distance-dependent attenuation model consistent with the log-distance path loss model widely adopted in WSN simulation studies [39]. Idle and state-report energy costs ($C_{\mathrm{idle}}=9.0$ J/h, $C_{\mathrm{sr}}=0.3$ J/step) are within the range reported for commercial WSN hardware such as the MICAz and TelosB platforms [40]. While the simulator does not model channel fading, packet collisions, or multi-hop routing, these simplifications are standard in the WSN scheduling literature [6,7] and isolate the scheduling decision from communication-layer effects, enabling a controlled comparison of policy performance. Validation of the integrated model against physical testbed data is identified as a priority for future work.

### 8.8. Scalability Considerations

**Asymptotic complexity of the guard layer.** At each scheduling step, the guard performs three operations whose costs are well defined in *N*. (i) The eligibility test (alive, sufficient battery, in detection radius) is an O(N) pass over the node set. (ii) The scoring function (10) requires computing the mean battery $\bar{E}$ across eligible nodes (O(N)) and then a single scoring pass (O(N)) to identify the highest-scoring eligible node, where Jain’s-fairness-equivalent load metrics are evaluated incrementally rather than over a quadratic candidate set. (iii) The action substitution and masking step is O(1) given the score. The aggregate per-step complexity is therefore Θ(N) in the number of nodes. There is no candidate-action enumeration that scales super-linearly in *N*: the guard scores each eligible node once and selects the maximum, rather than evaluating all N2 or $N^{2}$ pairs. Concretely, at N=30, the per-step overhead measured on a single CPU thread was <0.1 ms; extrapolating linearly under the Θ(N) bound, N=100 corresponds to approximately 0.3 ms and N=500 to approximately 1.5 ms per decision, both negligible relative to the hourly scheduling cadence and within the budget of resource-constrained embedded controllers (e.g., ARM Cortex-M class).

**Empirical validation regime and projected scaling beyond it.** The fixed-size wrapper supports up to $C_{\max}\times M_{\max}$ nodes with zero-padding and masking, avoiding retraining for smaller networks. As *N* increases beyond approximately 100 nodes, the policy network rather than the guard becomes the bottleneck: the action space grows large and sparse, the per-cluster observation aggregation loses granularity, and PPO sample efficiency degrades. For deployments at N=300−500, a hierarchical architecture is the natural extension: local controllers manage intra-cluster scheduling using the guard-enhanced PPO framework described here, while a coordination layer handles inter-cluster resource balancing and event routing. Each local controller operates over a tractable cluster size ($N_{\mathrm{local}}\leq 30$), preserving the regime where the proposed approach has been empirically validated, while the coordination layer can employ lightweight heuristics or a separate RL agent trained at the cluster-scheduling granularity. We note that simulating N=100 or N=500 end to end at the temporal resolution used here ($T_{\max}=8760$ steps, full-year horizon, PVGIS-driven solar yield) was computationally prohibitive within the present study’s resources; we therefore present the Θ(N) analysis and the hierarchical-architecture sketch in lieu of empirical large-scale validation and identify it as the priority for follow-on work.

## 9. Limitations and Future Work

The current work has several limitations that motivate future investigation. The system model assumes single-hop communication; extending the guard layer to multi-hop relay scoring is non-trivial. The guard layer provides empirical constraint enforcement but lacks formal safety guarantees—future work should explore control barrier functions or Lyapunov-based certificates for provable battery bounds. The evaluation considers up to 30 nodes; scalability to 100+ node networks requires hierarchical or federated learning architectures. While the sensitivity analysis (Section 7.8) demonstrates robustness across event rates, real seismic activity is spatially correlated and temporally non-stationary, and the PVGIS solar profile covers a single geographic location. The radio model assumes ideal communication without channel fading or packet loss. Finally, the current evaluation uses a single spatial topology per scale; whether policies generalise across topologies without retraining remains an open question that domain randomisation during training could address.

## 10. Conclusions

This paper has presented a safety-constrained reinforcement learning framework for transmission scheduling in energy-harvesting seismic wireless sensor networks. The integration of PPO with action masking and a runtime guard-layer safety filter demonstrates that hard safety constraints can be imposed on learned policies with minimal performance degradation. At 30 nodes, the guard-enhanced PPO achieves 99.46% transmission success and 66.47% node survival—a 58.3% improvement in survival over the highest-reward heuristic baseline (Closest)—while simultaneously outperforming unconstrained PPO on reward, transmission success, and survival. The guard layer’s scoring mechanism, combining battery headroom with load equity, provides an interpretable and configurable safety interface between the learned policy and the physical network. Sensitivity analysis across event rates confirms that these benefits are robust to operating conditions and not artefacts of the extreme stress-test scenario. The experimental analysis across three network scales identifies distinct operational regimes and quantifies the reward–survival trade-off that is fundamental to energy-constrained WSN scheduling. These results establish that safety-constrained RL is a viable and effective approach for long-term autonomous management of critical monitoring infrastructure.

## Figures and Tables

**Table 1:** Energy model parameters.

Parameter	Symbol	Value
Battery capacity	$E_{\max}$	15,000 J
Idle power	$P_{\mathrm{idle}}$	2.5 mW
Idle cost/step	$C_{\mathrm{idle}}$	9.0 J
Base TX cost	$C_{\mathrm{tx},\mathrm{base}}$	3.0 J
TX stress growth	$\gamma_{\mathrm{stress}}$	0.0015
Aging rate	$\gamma_{\mathrm{age}}$	$5\times 10^{-5}$
Panel efficiency	η	12%
Panel area	*A*	1.2 ${\mathrm{cm}}^{2}$
State-report scale	—	10% of TX cost
Peak harvest	$H_{\mathrm{peak}}$	49.8 J/h
Detection radius	$R_{\mathrm{detect}}$	250 km
Event probability	$p_{\mathrm{event}}$	0.9

**Table 2:** PPO training hyperparameters.

Parameter	Value
Learning rate α	$5\times 10^{-4}\to 5\times 10^{-6}$ (linear decay)
Network architecture	MLP [256, 256]
Batch size	256
Steps per rollout	8760 (one full episode)
Epochs per update	10
Entropy coefficient	0.02
Value function coefficient	0.5
Clipping parameter ϵ	0.2
Discount factor γ	0.99
GAE parameter λ	0.95

**Table 3:** Guard-layer load-balancing weight sensitivity at 30 nodes (RL+Guard, $p_{\mathrm{event}}=0.9$, 25 episodes). Metrics are bit-identical for α∈[0,0.5] in agreement with the rank-invariance derivation (12); the degeneracy at α=1.0 is empirically confirmed. Bold values denote the configuration adopted in the manuscript (α=0.2).

α	Survival (%)	Tx (%)	Reward	Load-Balance CV	Final Alive (Mean)
0.0	65.47	99.34	8280.22	1.332	19.64
0.1	65.47	99.34	8280.22	1.332	19.64
0.2	**65.47**	**99.34**	**8280.22**	**1.332**	**19.64**
0.3	65.47	99.34	8280.22	1.332	19.64
0.5	65.47	99.34	8280.22	1.332	19.64
1.0	62.80	99.26	8258.21	1.687	18.84

**Table 4:** Performance metrics across network scales (mean ± 95% CI, *t*-distribution, df=49, N=50 episodes). JFI values are point estimates averaged over episodes; per-episode CIs require full per-node battery distributions not retained in the summary pipeline. ^‡^

Scale	Policy	Reward	Tx Succ. (%)	Surv. (%)	Avg Batt. (%)	Load JFI ^‡^
10 nodes	Fixed ^†^	−1766±70	23.54	100.00	93.78	1.000
	Round-Robin	10,849±67	100.00	100.00	93.77	1.000
	Closest	11,770±70	100.00	100.00	93.77	1.000
	RL (PPO)	9937±65	100.00	100.00	93.76	1.000
	RL+Guard	11,278±68	100.00	100.00	93.77	1.000
15 nodes	Fixed ^†^	−2961±247	31.88	66.53	75.25	0.596
	Round-Robin	5013±290	95.70	36.53	89.56	0.399
	Closest	5961±328	95.08	34.80	91.20	0.394
	RL (PPO)	3038±324	96.74	54.13	78.90	0.186
	RL+Guard	4634±273	**97.58**	**62.93**	73.69	0.398
30 nodes	Fixed ^†^	−4059±191	22.71	69.20	75.62	0.669
	Round-Robin	7865±181	98.74	52.87	75.51	0.425
	Closest	8944±190	97.33	42.00	84.49	0.381
	RL (PPO)	7532±233	98.67	57.60	78.40	0.191
	RL+Guard	8393±186	**99.46**	**66.47**	74.31	**0.362**

^†^ Fixed JFI reflects a bimodal battery distribution (one depleted node, remainder full) rather than genuine load equity; see Section 7.4.

**Table 5:** Percentage improvement of RL+Guard over baselines (30 Nodes).

Baseline	ΔReward	ΔTx	ΔSurv.	ΔJFI
Fixed ^†^	+306.8%	+338.5%	−3.9%	—
Round-Rob.	+6.7%	+0.7%	+25.7%	−14.8%
Closest	−6.2%	+2.2%	+58.3%	−5.0%
RL (PPO)	+11.4%	+0.8%	+15.4%	+89.5%

^†^ Fixed JFI reflects bimodal battery distribution (one depleted node, remainder full) rather than genuine load equity; see Section 7.4.

**Table 6:** Sensitivity to event probability at 30 nodes (30 episodes per cell). *Surv. gap* columns give RL+Guard’s survival advantage in percentage points over the corresponding heuristic. The advantage grows monotonically with $p_{\mathrm{event}}$; no break-even exists in the realistic range. Italic rows are metric-panel subheadings; bold values indicate the proposed RL+Guard method in the survival-rate panel and the highest value in each row in the reward panel.

$p_{\mathrm{event}}$	Fixed	RR	Closest	RL	RL+Guard	Gap vs. RR	Gap vs. Cls
*Survival rate (%)*							
0.1	68.89	67.33	66.11	67.33	**68.78**	+1.45	+2.67
0.2	68.89	65.56	62.44	65.11	**68.56**	+3.00	+6.12
0.3	68.89	64.78	59.11	62.11	**68.22**	+3.44	+9.11
0.5	68.89	59.67	50.33	59.67	**67.44**	+7.77	+17.11
0.7	68.89	56.00	45.33	58.33	**67.33**	+11.33	+22.00
0.9	68.89	52.44	41.78	57.22	**66.22**	+13.78	+24.44
*Mean total reward*							
0.1	−1162	58	102	43	**218**	—	—
0.5	−2636	3952	**4394**	3529	4360	—	—
0.9	−4080	7895	**9034**	7472	8457	—	—
*Transmission success (%)*							
0.1	22.84	99.58	99.40	99.43	**99.64**	—	—
0.5	22.57	99.25	98.33	98.87	**99.55**	—	—
0.9	22.76	98.84	97.20	98.63	**99.44**	—	—

**Table 7:** Between-block variance for RL+Guard at 30 nodes, $p_{\mathrm{event}}=0.9$. Each block is an independent 50-episode draw; *stdev* is computed across the four block-level means.

Metric	Block 42	Block 142	Block 242	Block 342	Mean	Stdev
Survival rate (%)	66.47	65.27	66.33	62.47	65.13	1.86
Mean total reward	8393	8338	8226	7897	8214	222
Tx success (%)	99.46	99.38	99.35	99.26	99.36	0.08
Final alive nodes	19.94	19.58	19.90	18.74	19.54	0.56

**Table 8:** Empirical comparison at 30 nodes, $p_{\mathrm{event}}=0.9$, N=50 episodes. RL+Guard wraps the unconstrained PPO model with the runtime guard; Lagrangian-PPO replaces the guard with a soft constraint enforced by an adaptive multiplier. The Lagrangian formulation extracts marginally higher reward than unconstrained PPO but does not improve network survival; RL+Guard wins on every metric. Bold values indicate the best (winning) value in each column.

Method	Survival (%)	Tx Success (%)	Reward	Load-Balance CV
RL (unconstrained PPO)	57.60	98.67	7532	2.06
Lagrangian-relaxed PPO	56.60	98.15	7836	1.94
RL+Guard (proposed)	**66.47**	**99.46**	**8393**	**1.33**

## Data Availability

The simulation code and trained model weights used in this study are available from the corresponding author upon reasonable request.

## Abbreviations

The following abbreviations are used in this manuscript:

WSN	Wireless Sensor Network
RL	Reinforcement Learning
PPO	Proximal Policy Optimisation
GAE	Generalised Advantage Estimation
JFI	Jain’s Fairness Index
PVGIS	Photovoltaic Geographical Information System
MDP	Markov Decision Process
CPO	Constrained Policy Optimisation
